# Do people perceive benefits in the use of social prescribing to address loneliness and/or social isolation? A qualitative meta-synthesis of the literature

**DOI:** 10.1186/s12913-022-08656-1

**Published:** 2022-10-19

**Authors:** M. Liebmann, A. Pitman, Yung-Chia Hsueh, M. Bertotti, E. Pearce

**Affiliations:** 1 Division of Psychiatry, University College London, Maple House, 149 Tottenham Court Rd, London, W1T 7BN UK; 2 Institute for Connected Communities, University of East London, Water Lane, London, E15 4LZ UK

**Keywords:** Loneliness, Social isolation, Social prescribing, Wellbeing, Public health, Evaluation, Qualitative research

## Abstract

Social prescribing is a means by which clinical services can link individuals who have psychological, social and/or practical needs with non-clinical services within their local community. There is a lack of empirical evidence investigating whether social prescribing helps such individuals and which interventions are the most effective and accepted by them to address their loneliness. This meta-synthesis aimed to synthesise findings from qualitative studies exploring experiences of people (of any age) who participated in any social prescribing intervention aimed at loneliness and/or social isolation to ascertain whether they felt it helped address loneliness/isolation and the potential mechanisms by which this might occur. We conducted a systematic search of 5 electronic databases and 4 other databases that would yield grey literature in April 2021 to identify qualitative articles on this topic written in English or French. We assessed the quality of the included studies using recognised tools, and synthesised findings using the approach of thematic analysis. We identified 19 records analysed (e.g. journal articles) from 18 studies meeting inclusion criteria. Our analysis identified three themes: (1) increased sense of wellbeing (with six subthemes), (2) factors that engendered an ongoing desire to connect with others, and (3) perceived drawbacks of social prescribing. These themes illustrate the benefits and difficulties people perceive in social prescribing programmes addressing loneliness and social isolation, with an overall balance of more benefits than drawbacks in social prescribing participation. However, given the unhelpful aspects of social prescribing identified by some participants, greater thought should be given to potential harms. Moreover, further qualitative and quantitative research is needed to better understand mechanisms and effectiveness, and how different components of social prescribing might be best matched to individual participants.

**Supplementary Information:**

The online version contains supplementary material available at 10.1186/s12913-022-08656-1.

## Introduction

Loneliness is associated with a premature mortality risk of 26% [1] and other physical and mental health problems such as increased risk of coronary heart disease and stroke [2], depression, anxiety and suicidal ideation [3–6]. Feeling lonely or socially isolated can have harmful effects on an individual’s health that are comparable to those of smoking or obesity [7].

Loneliness is a global public health issue [1] and, in the UK, it has attracted considerable policy attention with the introduction of a governmental loneliness strategy in 2018 [8]. The COVID-19 pandemic has created an even greater need for action. Evidence suggests that the COVID-19 pandemic and lockdown measures were followed by increased rates of loneliness overall in the population [9–14], even several months after the pandemic occurred [15].

However, there is still considerable uncertainty about which interventions may be most appropriate and effective at reducing loneliness. For example, a meta-analysis of interventions to reduce loneliness in the general population showed that the strength of the evidence to support one-to-one support aimed at addressing maladaptive social cognitions was greater than that for interventions increasing opportunities for social interaction [16]. On the other hand, a review of systematic reviews concluded that although generally trial evidence of effectiveness was limited, group-based activities and support that provides social interaction appear to show some promise in addressing social isolation and loneliness [17].

One explanation for this apparent inconsistency may be that some research studies conflate the concepts ‘loneliness’ and ‘social isolation’, despite the two terms referring to different, though related, phenomena. *Loneliness* is defined as a perceived mismatch between one’s desired and actual social relationships [18] where an individual sees themself as socially isolated even if they have opportunities to engage socially [1]. *Social isolation* refers to the objective absence or paucity of social contacts and interactions [19]. However, this inconsistency could also possibly be due to the use of different measures to capture loneliness and social isolation [20]. Currently we do not know whether some interventions work better for loneliness than social isolation or vice versa.

One way of tackling loneliness and promoting social connections is through social prescribing (SP) interventions [21]. Although primarily social interventions, the variety and nature of the activities prescribed mean that they could have psychological effects such as to enhance social skills, improvement in perceived social support, and address impaired social cognition, as well as their primary aim of increasing opportunities for social interaction [16]. The UK Social Prescribing Network describes SP as “enabling healthcare professionals (e.g., general practitioners (GPs)) to refer patients to a link worker, to co-design a non-clinical social prescription to improve their health and wellbeing” [22]. As the title suggests, link workers represent the link between referring clinicians, patient and local voluntary or statutory community resources [23, 24]. Such resources include art-based activities, walking clubs, communal gardening, advice services and exercise classes [23, 25, 26]. SP programmes are being progressively implemented across the UK [21]. Indeed, in 2019 the *NHS Long Term Plan* made the commitment to train over 1,000 social prescribing link workers by the end of 2020/21, whose aims are to develop tailored plans with the referred patient and connect them to local groups and support services with the ultimate aim of reaching 900,000 people across England by 2023/24 [26]. Additionally, in response to the Covid-19 pandemic, forms of social prescribing in the UK have been scaled up and adapted to meet the need for online/blended provision [24].

Despite the pace of this implementation there are few randomised controlled trials (RCTs) investigating the effectiveness of SP in alleviating loneliness, and those trials have been appraised as low quality [21, 27, 28]. Instead, we rely on evidence predominantly from observational studies providing weak evidence (mainly due to methodological problems) to support positive impacts of SP on loneliness [27–29], although in some cases no positive impacts have been found [21]. However, none of these reviews have examined the mechanisms underpinning the relationship between loneliness and social prescribing, which is particularly important to understand given the uncertainty about the most suitable types of intervention (e.g., group or one-two-one) surrounding interventions to reduce loneliness. Additionally, no reviews have been conducted of the qualitative literature describing perceptions of the effects of SP in relation to loneliness and social isolation. Given these uncertainties over which are the best types of SP intervention to reduce loneliness, the growing national policy importance of social prescribing, and the uncertainty over how social prescribing may contribute to reduce loneliness, this paper aims to address these gaps in the literature by conducting a qualitative meta-synthesis of the literature investigating the acceptability and perceived effectiveness of SP on loneliness and/or social isolation from the perspective of the participants. Studies needed to include data exploring one or both these outcomes to be included in our review. We aimed to investigate both loneliness and social isolation due to the difficulty in distinguishing between them in the primary literature. Gaining a better understanding of the perceived acceptability, benefits and harms of SP on loneliness may help in further intervention development, including the use of more appropriate measurement tools for trials.

## Methods

We conducted a qualitative meta-synthesis of the literature capturing participant’s experience of SP interventions to address loneliness and/or social isolation. A meta-synthesis is a systematic approach used to search, screen, extract, and code qualitative data [30]. The approach involves combining findings across different qualitative studies to ascertain patterns and common themes within a particular topic as well as to enhance the understanding of evidence-based interventions [31]. Meta-synthesis represents a research team’s interpretation of original data and analysis from the constituent empirical qualitative studies [32], which must be analysed in sufficient detail to maintain the integrity of each study [33]. Qualitative syntheses are acknowledged as useful tools for analysing participants’ meanings, experiences and perspectives, both deeply (thanks to the qualitative approach) and widely (thanks to the combination of articles from different backgrounds and participants) [30]. We followed the established six step meta-synthesis approach: 1) defining the research question and selection criteria, 2) driving the selection of the studies, 3) conducting the quality assessment of the studies, 4) extracting and presenting the formal data, 5) directing the data analysis and 6) writing the synthesis [30], as used previously by our team when investigating experiences of loneliness in young people with depression [34].

### Inclusion and exclusion criteria

Table 1 shows the eligibility criteria.

**Table 1:** Inclusion and exclusion criteria

Key Concept	Inclusion Criteria	Exclusion Criteria
Participants	Studies that sampled participants of any age describing experiences of loneliness and/or social isolation	Studies that sampled participants without a history of loneliness and/or social isolation Participants reporting cognitive impairments (e.g., dementia, psychosis)
Intervention	Studies that included any well-defined intervention considered as a SP intervention, delivered individually or by groups through any means (e.g., face-to-face, internet, telephone) regardless of the duration or number of treatment sessions	Studies that included any intervention not considered a SP intervention
Study design	Descriptions of loneliness and service users’ views about the use of SP to address their loneliness and/or social isolation. Studies that analysed data from focus groups, semi-structured interviews and textual data (e.g., personal written account) were included For mixed methods studies, only the qualitative data were extracted	Any quantitative research designs
Language	Articles written in English or French	Articles written in languages other than English or French

### Search strategy

The team’s protocol was discussed with a researcher with relevant lived experience of loneliness. Thus, initial search terms were based on the academic literature and combined expertise of the team, and then the lived experience researcher was asked to identify any gaps. No new terms were added to those identified for the academic literature. We pre-registered the protocol on PROSPERO (registration number: CRD 42,021,246,421) and followed PRISMA guidelines to conduct the review [35].

We developed search terms (see Supplementary Material for the exact search terms used) to capture loneliness, social prescribing schemes (including terms related to wellbeing coordinators and community navigation, to ensure the search was comprehensive) and qualitative research. We included any article with an intervention considered as SP according to The UK Social Prescribing Network’s definition (see introduction). This definition includes the presence of a link worker as the key necessary element of social prescribing. We amended our PROSPERO protocol after pilot searches but before the final searches to reflect the need to include social isolation as a search term as many research studies did not distinguish the related terms “loneliness” and “social isolation”.

We searched five electronic databases (Scopus, Web of Science Core Collection, Medline via Ovid, PsycInfo via Ovid and Embase via Ovid) from inception up to April 2021 to identify qualitative articles published in English or French.

We also searched databases that would yield grey literature i.e. articles and reports (including theses, NGO reports, government policies) that have not been published in a peer-reviewed journal: Google Scholar, and the Networked Digital Library of Theses and Dissertations, as well as the King’s Fund Library’s website, and the Nuffield Trust’s website using specific search terms (see Supplementary Material for the exact search terms used to search grey literature). Additionally, a mix of peer-reviewed articles and non peer-reviewed evaluation reports were provided by one co-author (MB); an expert academic steering group member of the Social Prescribing Network who had collated these from previous research studies. No date restrictions were used. The electronic database searches were supplemented by hand-searching the reference lists of any eligible studies to reduce the chance of missing relevant studies.

### Screening

All searches of the peer-reviewed literature used all the available fields including title, abstract, and key words. All studies were retrieved by ML and all titles/abstracts were screened for eligibility by ML using the software EndNote X9; full texts of potentially eligible papers were then screened by ML. A second reviewer (YH) independently screened a randomly assigned 20% of full text studies to establish agreement over eligibility criteria, and any disagreements were resolved by discussion between the two reviewers, seeking input from a third author where necessary.

### Quality assessment

The Critical Appraisal Skills Programme (CASP), a 10-item quality assessment tool for qualitative research was used to assess all eligible studies [36]. This tool appraises ten areas: clarity of research aims, appropriateness of qualitative methodology, research design, recruitment strategy, data collection and researcher reflexivity, consideration of ethical issues, appropriateness of data analysis, clarity of stated findings and value of the research [36]. Ratings range from 1 to 10 (1 = low quality and 10 = high quality) for peer-reviewed articles using CASP and from 1 to 6 (1 = low and 6 = high) for non peer-reviewed records using AACODS. Studies meeting eligibility criteria were included in the review regardless of their quality, given that there may be relevant themes in studies despite poor quality of the study methods. The meta-analytic approach looks for convergent themes across different studies, rendering the quality of individual studies less relevant [30, 37]. In addition, the evaluation and critical appraisal of non-academic papers and grey literature was conducted using the AACODS checklist [38]. Quality appraisal of all studies was conducted by one researcher (ML) and 20% were appraised independently by a second reviewer (YH).

### Data extraction and data synthesis

One researcher (ML) identified any text relating to loneliness or social isolation within the results section of included studies (quotes and/or authors’ interpretations) and imported this into NVivo, a qualitative data software package [39].

One researcher (ML) then coded the full dataset, and one researcher (YH) checked the codes against the data from four (equivalent to 20% of the data) randomly allocated studies. Coding involved familiarisation with the data, examination of existing themes in each article against the aims of the meta-synthesis, and generation of initial codes regardless of the existing descriptive labels given by the original study authors. Team discussions were used to collate codes into overarching themes and subthemes, in a process of iterative development. Identified themes and subthemes were then reviewed and refined to ensure they were meaningful and clearly distinct from each other. Finally, themes were further refined and renamed by identifying their ‘essence’ and determining the element of the data each theme captures. This final taxonomy of analytical themes was used to present quotes providing a valid and varied account of the data within and across the themes.

### Reflexivity and external validity

Any synthesis of qualitative studies is not a mere summary of findings of the included studies, but rather a re-conceptualizing and interpretation of findings to develop new insights that would not be achieved in any individual empirical study [40]. Therefore, the reviewers’ interpretations and understanding of the data are likely to influence the process of synthesis [41]. The multidisciplinary nature of our research team (combining clinical psychiatry, epidemiology, anthropology, human geography, sociology, and psychology) incorporated multiple perspectives providing a more holistic picture, which in turn enhanced external validity. The presence of an independent reviewer reduced bias in the screening and quality appraisal process. Team discussions to support the iterative development of the codes and themes included consideration of reflexivity, which was enhanced through gaining input from a researcher with relevant lived experience.

## Results

**Fig. 1:**
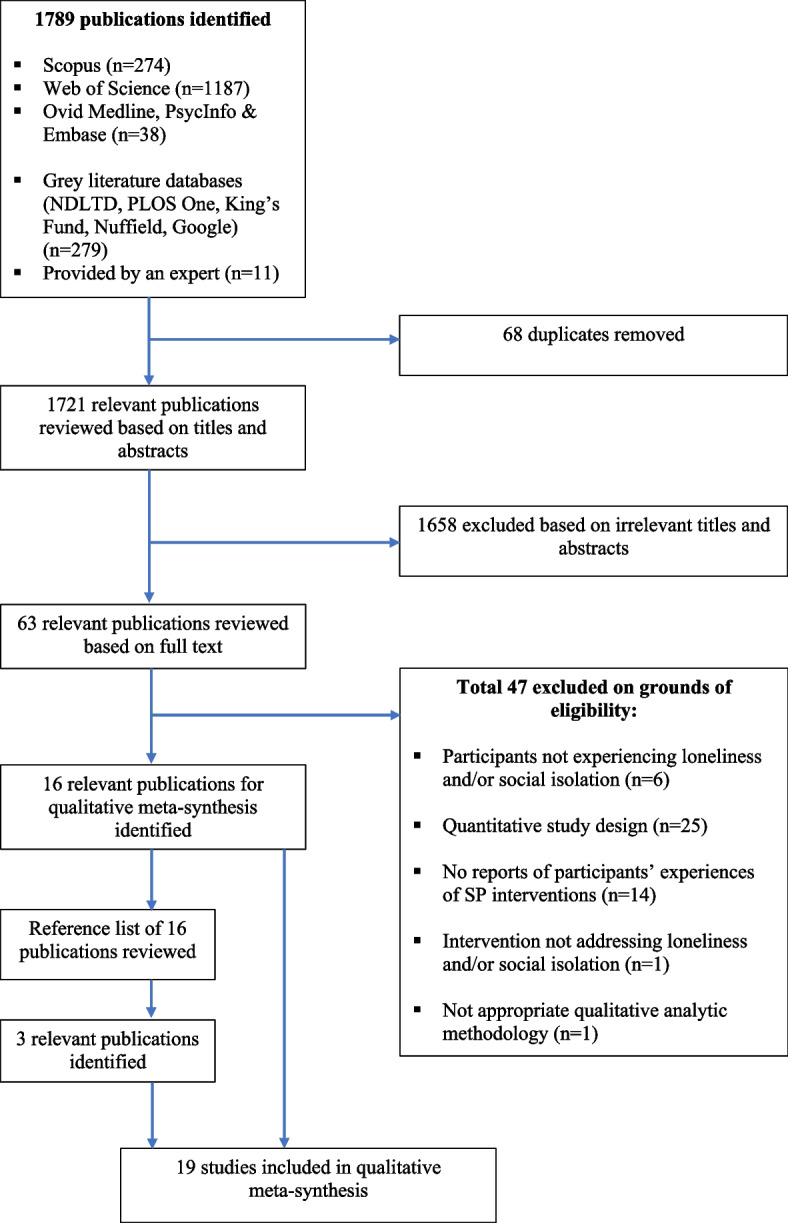
PRISMA flow chart

The searches yielded 1499 peer-reviewed articles and 290 non peer-reviewed records; 19 eligible articles were included in our review (17 from peer-reviewed literature and two from grey literature) (Fig. 1). These reported findings from 18 studies: two papers reported findings from the same sample, but were both included as they had distinct aims [42, 43]. Characteristics and quality appraisal of each study are given in Table 2 including the original relevant themes. The Cohen’s kappa statistic for data extraction was 0.83, indicating strong inter-rater agreement [44].

**Table 2:** Study characteristics

Author(s), year of publication, country of study	Sample size	Population studied	Aims relevant to our research questions	Data collection	Analysis	Findings (themes related to our research questions only)	Quality appraisal CASP (published studies): 1–10 AACODS (unpublished studies): 1–6
**(Alliance for Healthier Communities, 2020) **[45]**, Canada** **Unpublished literature**	1101 (63.9% female; 32.7% male; 3.4% other)	Aged 18–81 + . People coming from 11 Clinical Commissioning Groups representing a diverse mix of urban, rural, Northern, and Francophone communities	Understanding the contextual factors, facilitators, challenges, and impact of social prescribing	Focus groups using a semi-structured interview format (face-to-face) (*number of focus groups not stated*) Electronic medical records (EMR) data extraction	Thematic analysis	1)Improved mental wellbeing 2)Greater capacity to self-manage health 3)Decreased loneliness, increased sense of connectedness and belonging	5/6 Y^a^ – Authority Y – Accuracy N^b^ – Coverage Y – Objectivity Y – Date Y – Significance
**(Blickem et al., 2013) **[46]**, UK**	34 males and females (% not stated)	People living with long-term health conditions (e.g., cardiac issues, diabetes, etc.) and who are often isolated, recruited from health-related support groups and community centres offering a variety of activities (e.g., exercise, hobbies, interests) relevant to health or wellbeing	Exploring the meaning and role of the community and voluntary sector for people with physical and mental long-term health conditions	Focus groups (*n* = *5*) (face-to-face)	Thematic analysis	1)Isolation, safety and linking to support 2)The group’s power to normalize the problems of chronic illness 3)Reciprocal communities	9/10 Y—Clear aims Y—Appropriate qualitative methodology Y—Appropriate research design Y—Appropriate data collection Y—Appropriate recruitment strategy N—Considered reflexivity appropriately Y—Ethical considerations addressed Y—Rigorous data analysis Y—Clear statement of findings Y—Value of research
**(Cheetham et al., 2018) **[47]**, UK**	25 (15 female & 10 male)	Aged 34–71. People experiencing a variety of psychosocial and economic issues (e.g., loneliness, social isolation, significant mental health concerns, anxiety and depression, stress, caring responsibilities, family worries, grief, loss, and bereavement)	Exploring service users’ experiences and understanding the factors influencing service user engagement and the ‘active ingredients’ that prompt change	In-depth semi-structured interviews (face-to-face) Short Warwick Edinburgh Mental Wellbeing Scale (SWEMWBS)	Thematic analysis	1)What helped individuals’ progress towards meeting self-identified goals? 2)What helped communities?	6/10 Y—Clear aims Y—Appropriate qualitative methodology Y—Appropriate research design Y—Appropriate recruitment strategy Y—Appropriate data collection N—Considered reflexivity appropriately N—Ethical considerations addressed N—Rigorous data analysis Y—Clear statement of findings N—Value of research
**(Fortune et al., 2021) **[48]**, Canada**	19 (16 female & 3 male)	Mostly aged 65 + . People recruited from three art hives (‘public home places’) who differ in terms of cultural background, income levels and health status	Exploring the perceptions of belonging and the ways in which the involvement in the intervention contributed to the sense of belonging	Focus groups (*n* = *3*) and interviews (face-to-face)	Thematic analysis	1)Needing social spaces 2)Being welcomed into a non-judgemental Space 3)Coming for more than just the art 4)Creating a shared space	8/10 Y—Clear aims Y—Appropriate qualitative methodology Y—Appropriate research design N—Appropriate recruitment strategy Y—Appropriate data collection N—Considered reflexivity appropriately Y—Ethical considerations addressed Y—Rigorous data analysis Y—Clear statement of findings Y—Value of research
**(Frerichs et al., 2020) **[49]**, UK**	19 (14 female & 5 male)	Aged 18–70. People experiencing severe depression and/or anxiety, and loneliness	Exploring service users’ experiences to understand what affects people’s ability to embrace loneliness interventions	Semi-structured interviews (face-to-face, except one with telephone)	Thematic analysis and narrative analysis	1)Desire to connect with others 2)Individual’s social confidence 3)Finding something meaningful 4)Accessible resources locally 5)Relationship with community navigator	7/10 Y—Clear aims Y—Appropriate qualitative methodology N—Appropriate research design Y—Appropriate recruitment strategy Y—Appropriate data collection N—Considered reflexivity appropriately N—Ethical considerations addressed Y—Rigorous data analysis Y—Clear statement of findings Y—Value of research
**(Giebel et al., 2020) **[50]**, UK**	13 (9 female & 4 male)	Aged 44–84. Individuals at risk of feeling lonely and isolated with “low levels of mental health needs”	Evaluating services users’ experiences and impact of whether accessing Community Connectors (social support service) on loneliness	Semi-structured interviews (face-to-face)	Thematic analysis	1)Structured supportive service 2)Reconnecting with community	6/10 Y—Clear aims Y—Appropriate qualitative methodology N—Appropriate research design Y—Appropriate recruitment strategy Y—Appropriate data collection N—Considered reflexivity appropriately N—Ethical considerations addressed N—Rigorous data analysis Y—Clear statement of findings Y—Value of research
**(Greaves & Farbus, 2006) **[51]**, UK**	18 (11 female & 7 male)	Aged 50 + . People whose lives have changed or are about to change, or people with time on their hands or who find it difficult to keep in touch with the local community	Identifying the range and nature of impacts of the intervention on participants	Semi-structured interviews and focus groups (*n* = *1*) (face-to-face) SF12 Health Quality of Life	Thematic analysis	1)Psychological and social benefits 2)Factors mediating the impact of Upstream	8/10 Y—Clear aims Y—Appropriate qualitative methodology Y—Appropriate research design Y—Appropriate recruitment strategy Y—Appropriate data collection N—Considered reflexivity appropriately N—Ethical considerations addressed Y—Rigorous data analysis Y—Clear statement of findings Y—Value of research
**(Greenfield & Mauldin, 2017) **[52]**, USA**	41 (29 female & 12 male)	Aged 60 + . More than half the participants lived alone	Exploring the meaning of the contact with others at community activities offered by the intervention	In-depth semi-structured interviews (face-to-face)	Grounded theory	1)Personal need for additional social activity 2)Health status 3)Relationships with staff 4)Appeal of the other attendees 5)Experiencing a social environment 6)Replicating existing contacts 7)Sense of community 8)Activity-based friendships 9)Independent friendships 10)Perceptions of programme attendees	7/10 Y—Clear aims Y—Appropriate qualitative methodology N—Appropriate research design Y—Appropriate recruitment strategy Y—Appropriate data collection N—Considered reflexivity appropriately N—Ethical considerations addressed Y—Rigorous data analysis Y—Clear statement of findings Y—Value of research
**(Hemingway & Jack, 2013) **[53]**, UK**	82 (over 80% female)	Average aged 80. 80% of the participants lived alone	Exploring perceptions of the impact of attending the clubs on wellbeing/mental-physical health	Interviews and focus groups (*number of focus groups not stated*) (face-to-face)	Interpretative analysis, inductive content analysis and constant comparative strategy	1)Feeling isolated 2)Friendship	10/10 Y—Clear aims Y—Appropriate qualitative methodology Y—Appropriate research design Y—Appropriate recruitment strategy Y—Appropriate data collection Y—Considered reflexivity appropriately Y—Ethical considerations addressed Y—Rigorous data analysis Y—Clear statement of findings Y—Value of research
**(Kellezi et al., 2019) **[54]**, UK** **(Study 1 in the article)**	19 (12 female; 1 male; 1 prefer not to say)	Aged 29–85. Individuals referred to a SP pathway for weight loss reasons and support for multiple/complex needs including loneliness	Investigating the degree to which the patients recognise experiences of social (dis) connection and appreciate the effects of these experiences, as well as SP’s potential to remedy their issues	In-depth semi-structured interviews (face-to-face)	Thematic analysis	1)Patients’ perspective: relationship with link workers/health coaches and building social connections	7/10 Y—Clear aims Y—Appropriate qualitative methodology N—Appropriate research design Y—Appropriate recruitment strategy Y—Appropriate data collection N—Considered reflexivity appropriately N—Ethical considerations addressed Y—Rigorous data analysis Y—Clear statement of findings Y—Value of research
**(Kharicha et al., 2017) **[55]**, UK**	28 (18 female & 10 male)	Aged 65 + . Participants identified as lonely (either self-identified or scored as lonely on a validated scale)	Exploring the perspectives of community dwelling lonely older people about seeking support for loneliness from primary and community-based services and the features of these services which informed their views	Interviews (face-to-face) De Jong Gierveld Loneliness Scale	Thematic analysis	1)Could befriending be for me? 2)‘Social groups’ are for others 3)Having a common interest 4)Dealing with loneliness privately	8/10 Y—Clear aims Y—Appropriate qualitative methodology Y—Appropriate research design Y—Appropriate recruitment strategy Y—Appropriate data collection N—Considered reflexivity appropriately N—Ethical considerations addressed Y—Rigorous data analysis Y—Clear statement of findings Y—Value of research
**(MacLeod, Skinner, Wilkinson, & Reid, 2016) **[56], **Canada**	8 (6 female & 2 male)	Aged 65–95. Participants were cognitively well but isolated	Exploring the experience of older adults who engage in expressive arts	Field notes, debriefing meeting minutes and audio logs	Thematic analysis and narrative analysis	1)Relationships 2)Personal development 3)Created meanings	7/10 Y—Clear aims Y—Appropriate qualitative methodology N—Appropriate research design Y—Appropriate recruitment strategy N—Appropriate data collection N—Considered reflexivity appropriately Y—Ethical considerations addressed Y—Rigorous data analysis Y—Clear statement of findings Y—Value of research
**(Moffatt et al., 2017) **[42]**, UK**	30 (14 female & 16 male)	Aged 40–74. Individuals had more than one long-term condition (e.g., diabetes, asthma, coronary heart disease, obstructive pulmonary disease, heart failure, epilepsy, osteoporosis), had physical and mental health issues, low confidence and social isolation	Capturing the experiences of patients engaged in the intervention in its first 14 months of operation and identifying the impact of the programme on health and wellbeing	Semi-structured interviews (face-to-face)	Thematic analysis	1)Positive impact of the link worker social prescribing programme (health-related behaviours; mental health; long-term condition management)	7/10 Y—Clear aims Y—Appropriate qualitative methodology N—Appropriate research design Y—Appropriate recruitment strategy Y—Appropriate data collection N—Considered reflexivity appropriately N—Ethical considerations addressed Y—Rigorous data analysis Y—Clear statement of findings Y—Value of research
**(Nordin et al., 2020) **[57]**, Sweden**	7 (5 female & 2 male)	Aged 79–94. Older adults who were receiving home care to meet social needs and/or experiencing loneliness. All were widowed and living alone	Exploring the perceptions and experiences of community-dwelling older adults with regard to aspects related to social participation in the specific context of living with support from home care services	Semi-structured interviews (face-to-face)	Content analysis	A.the ME: doing what I like: 1)Cultivating interests 2)Navigating occupations B.the WE: seeing and being seen by my people: 1)Being in a circle of significant people 2)Having practical and social needs seen, 3)Feeling equality and mutuality	10/10 Y—Clear aims Y—Appropriate qualitative methodology Y—Appropriate research design Y—Appropriate recruitment strategy Y—Appropriate data collection Y—Considered reflexivity appropriately Y—Ethical considerations addressed Y—Rigorous data analysis Y—Clear statement of findings Y—Value of research
**(Todd, 2017) **[58]**, UK** **Unpublished literature**	12 (7 female & 5 male)	Aged 64–84. Participants self-identifying as lonely or socially isolated	Exploring a large-scale social prescribing scheme’s use of museums to address the psychological wellbeing of socially isolated older people	Semi-structured interviews (face-to-face or by telephone) and diary notes UCLA Loneliness Scale Warwick Edinburgh Mental Wellbeing Scale (WEMWBS)	Grounded theory	A.Interacting social context 1)Evaluating self and others 2)Getting to know people 3)Social engagement 4)Sharing experiences B.Museum as enabler 1)container and provider 2)New experiences 3)Role of facilitator 4)Engaging with artefacts and arts C.Individual journey 1)Emotion D.Relational processes 1)Judging others 2)Influence of others	6/6 Y – Authority Y – Accuracy Y – Coverage Y – Objectivity Y – Date Y – Significance
**(Van De Venter & Buller, 2015) **[59]**, UK**	6 (3 female & 3 male)	Aged 25–59. People with mild-to-moderate mental health problems	Exploring participants’ process of change and experiences of the group activities	Individual interviews (face-to-face) Warwick Edinburgh Mental Wellbeing Scale (WEMWBS)	Thematic analysis	1)Differences by gender: normalizing emotions 2)Differences by ethnicity: the importance of breaking social isolation	6/10 Y—Clear aims Y—Appropriate qualitative methodology N—Appropriate research design N—Appropriate recruitment strategy Y—Appropriate data collection N—Considered reflexivity appropriately N—Ethical considerations addressed Y—Rigorous data analysis Y—Clear statement of findings Y—Value of research
**(Wildman et al., 2019) **[43]**, UK**	24 (11 female & 13 male)	Aged 40–74. Individuals had more than one long-term condition (e.g., diabetes, asthma, coronary heart disease, obstructive pulmonary disease, heart failure, epilepsy, osteoporosis), had physical and mental health issues, low confidence and social isolation	Exploring service-users’ experiences of social prescribing, one to two years after their initial involvement to the intervention	Semi-structured interviews (face-to-face)	Grounded theory and thematic analysis	1)The importance of the service user/link worker relationship 2)Making and maintaining progress in behaviour change and LTC self-management	8/10 Y—Clear aims Y—Appropriate qualitative methodology N—Appropriate research design Y—Appropriate recruitment strategy Y—Appropriate data collection N—Considered reflexivity appropriately Y—Ethical considerations addressed Y—Rigorous data analysis Y—Clear statement of findings Y—Value of research
**(Wood et al., 2021) **[60]**, UK**	18 (10 female; 7 male; 1 prefer not to say)	Aged 18 + . People with both physical and mental health issues	Exploring the mechanisms that facilitate engagement and positive outcomes with SP intervention among people with multiple health condition and social needs	Interviews (face-to-face) and one workshop	Realist evaluation/analysis	1)Social isolation	8/10 Y—Clear aims Y—Appropriate qualitative methodology Y—Appropriate research design Y—Appropriate recruitment strategy Y—Appropriate data collection N—Considered reflexivity appropriately N—Ethical considerations addressed Y—Rigorous data analysis Y—Clear statement of findings Y—Value of research
**(Woodall et al., 2018) **[61]**, UK**	26 (12 female & 14 male)	Aged 16–94. Many participants reported feeling socially isolated prior to engagement with the service	Exploring referral pathway, activities undertaken, perceived outcomes and benefits from the service, interaction with primary care services since enrolment and recommendations for service improvement	Semi-structured interviews (by telephone) Warwick Edinburgh Mental Wellbeing Scale (WEMWBS) EQ-5D Campaign to End Loneliness Measurement Tool	Thematic analysis	1)Wellbeing 2)Social networks 3)Use of general practitioners’ services 4)The attributes of the Wellbeing Coordinator	7/10 Y—Clear aims Y—Appropriate qualitative methodology Y—Appropriate research design N—Appropriate recruitment strategy Y—Appropriate data collection N—Considered reflexivity appropriately N—Ethical considerations addressed Y—Rigorous data analysis Y—Clear statement of findings Y—Value of research

^a^*Y* = *Yes*

^b^*N* = *No*

Of the six studies that were published during or after the COVID-19 pandemic, four of them [49, 50, 57, 60] collected data between 2017 and 2018. The remaining two studies [45, 48] did not specify the year of data collection, therefore we assumed that data were collected beforehand.

### Characteristics of included studies

The sample sizes ranged from 6 to 1101 participants (median = 19), with a total number of 1506 participants across all 18 studies. Participants’ ages ranged from 18 to 95 years, and the mean proportion of female participants (in studies specifying gender) was 63%. Two studies did not specify participants’ ages [46, 60] and one study did not specify participants’ gender [46]. Over half (53%) of studies sampled older adults only, making it harder to explore age patterning of themes. Loneliness was measured either using standardised quantitative measures (11%) such as the Warwick Edinburgh Mental Wellbeing Scale (WEMWBS), the Short Form 12 (SF12) Quality of Life, the de Jong Gierveld Loneliness Scale, and the UCLA Loneliness Scale, or directly by participants self-reporting being lonely/socially isolated (67%), or in both ways (28%). Details of the measures used are given in Table 2.

In 12 papers only a proportion of the sample (at least 50% of each) reported feeling lonely or socially isolated, with the remaining participants experiencing long-term physical or mental health conditions but not explicitly reporting that they were lonely/isolated [42, 45–49, 51–54, 59, 60].

Dates of publications ranged from 2006 to 2021. The majority (72%) of the 18 original studies sampled participants in the UK, three in Canada, one in the United States and one in Sweden. Only English language studies were retrieved. Overall 17 articles were published in peer-reviewed journals, one paper was a government report [45], and one article was an unpublished PhD thesis [58].

The SP interventions investigated included health-related support groups, community gardening, arts and recreational activities, exercise classes, luncheons clubs, welfare rights advice, and many others. Further descriptions of each SP intervention are presented in Table 3.

**Table 3:** Description of the SP interventions

Author(s), year of publication, country of study	Target population	Outcome(s) (loneliness and/or social isolation)	Details of delivery	Description of the SP interventions
**(Alliance for Healthier Communities, 2020) **[45]**, Canada** **Published report for 3**^**rd**^** sector**	- Diverse mix of people with social and medical complexities, e.g., living on low income or social assistance, newcomers, individuals with chronic conditions, mental health issues, or comorbidities, people from population groups facing the highest health disparities in Ontario – Black and racialized people, Indigenous people, those from Two Spirit (2S) and LGBTQ + , and Francophones	Loneliness and social isolation	- Face-to-face delivery - Group activities - 11 Community Health Centers (CHCs) developed a SP initiative within their community - Length of intervention: 18 months	*RX: Community* is a social prescribing pilot research project which consists of 11 Community Health Centers (CHCs). These CHCs offer activities such as navigating housing and employment services, communal dining, exercise groups, coffee clubs, community gardens, sing-alongs, knitting classes, card game groups, pole walking, friendly visiting, arts classes, etc
**(Blickem et al., 2013) **[46]**, UK**	- People with long-term health conditions (LTCs)	Social isolation	- Face-to-face delivery - Group activities - Length of intervention: not stated	Community centers and health-related support groups (e.g., cardiac support group, diabetes support group, sugar group) offering activities such as exercise, hobbies, and interests. The provided activities or services were relevant to participants’ health and wellbeing
**(Cheetham et al., 2018) **[47]**, UK**	- People from deprived local communities - People with long-term health conditions - People with poorer healthy life expectancy	Social isolation	- Face-to-face delivery - Individual sessions (up to 12) and group activities - Length of intervention: 12 weeks	*Live well Gateshead (LWG)* aims to promote health and wellbeing through a combination of tailor made, lifestyle interventions for individuals, groups, families and communities. Services provided by Local Authorities and NHS staff include 1:1 session with wellness coaches, group work, smoking cessation, weight management, dietary and healthy eating advice, physical activity pathways, mental health and emotional wellbeing interventions, alcohol brief interventions, signposting and accompanied referral to specialist agencies, such as welfare rights and housing advice
**(Fortune et al., 2021) **[48]**, Canada**	- Older adults with diverse cultural background, income levels and health status	Loneliness and social isolation	- Face-to-face delivery - Group activities - Length of intervention: 4 months	Art Hives are defined as free inclusive community art spaces and designed as ‘public home places’. In these places, participants were encouraged to make art together
**(Frerichs et al., 2020) **[49]**, UK**	- People severely anxious and depressed	Loneliness	- Face-to-face delivery - Individual sessions (up to 10) and group sessions - Length of intervention: 2 years	The *Community Navigatior programme* includes receiving support from a ‘Community Navigator’ based in secondary mental health services who will help service users to increase social contact, participation in social activities and community engagement, with the aim of reducing feelings of loneliness. Besides, group sessions will take place where participants are encouraged to share information about local resources and social groups
**(Giebel et al., 2020) **[50]**, UK**	- People (aged 18 and above) at risk of feeling lonely and isolated, with low levels mental health needs - People from any socio-economic background	Loneliness and social isolation	- Face-to-face delivery - Individual sessions and group activities - Length of intervention: 14 weeks	The *Community Connectors service* is a structured support service that enables access to local support through a range of early intervention and prevention services such as luncheon clubs, debt awareness, social activities, befriending, foodbanks, as well as commissioned services
**(Greaves & Farbus, 2006) **[51]**, UK**	- People from their 50 s onwards, whose lives may have changed or are about to change in some way (perhaps through retirement, moving home, age or illness), or people with time on their hands, or who might find it difficult to keep in touch with the local community - People with no mental or physical health problems	Loneliness and social isolation	- Face-to-face delivery and telephone contact with the LWs - Individual home sessions (only for participants with severe mobility problems) and group activities - Length of intervention: 1 year	*The Upstream Healthy Living Centre* is a community-based intervention which provides participants with programmes of creative, exercise and/or cultural activities, with an emphasis on social interaction. Offered activities include painting, print making, creative writing, reminiscence/living history, Tai Chi, movement/gentle exercise, computing, pottery, exploring sound and music, various craft work activities, quilting, falls awareness education, singing, hand bells, Walk and Talk groups, cookery, books clubs, and hearing school children read
**(Greenfield & Mauldin, 2017) **[52]**, USA**	- Older adults	Social isolation	- Face-to-face delivery - Group activities - Length of intervention: not stated	*Naturally Occurring Retirement Community Supportive Service (NORC) Programs* seek to promote health and wellbeing of older adults ageing in place in their own homes and communities. They are composed of a variety of types of community activities, including socio-recreational (e.g., yoga classes, luncheons and trips), educational (e.g., discussion groups and informational sessions) and civic (e.g., advisory council meetings, meetings with local government officials and intergenerational volunteering)
**(Hemingway & Jack, 2013) **[53]**, UK**	- Older adults	Loneliness and social isolation	- Face-to-face delivery - Group activities (once a week for 2 h) - Length of intervention: 3 years	The friendship clubs promote wellbeing for older people and offer several activities such as card games, outings, information giving sessions, physical exercise, guest speakers and entertainers, as well as informal conversation over a tea and cake
**(Kellezi et al., 2019) **[54]**, UK** **(Study 1 in the article)**	- People with chronic illness who are experiencing loneliness	Loneliness and social isolation	- Face-to-face delivery - Individual sessions and group activities - Length of intervention: 8 weeks	The SP pathway aims to increase patients’ illness self-management, address their psychosocial and health needs, and through this to reduce primary healthcare usage. To do so, patients are offered either one-to-one support meetings, or group activities that meet their needs
**(Kharicha et al., 2017) **[55]**, UK**	- Community dwelling lonely older adults aged 65 and over (recruited from 5 NHS primary care practices)	Loneliness and social isolation	- Face-to-face delivery - Individual sessions (primary based services) and group activities - Length of intervention: not stated	The SP intervention consists of primary and community-based services that offer both one-to-one and group-based support such as lunch clubs, coffee mornings, exercise groups, hobby-based activities, etc
**(MacLeod et al., 2016) **[56]**, Canada**	- Isolated older rural adults	Social isolation	- Face-to-face delivery - Individual sessions (*n* = 10) - Length of intervention: 10 weeks	*Visible Voices: Senior Connecting with Seniors through Expressive Arts Making* sought to address rural social isolation through the intervention of in-home volunteer-based expressive arts. Socially isolated older adults were matched one-to-one with older adult volunteers to conduct in-home, individual, intermodal art-making activities in their dyads. The volunteers included retired artists, teachers, and nurses, among others
**(Moffatt et al., 2017) **[42]**, UK**	- People aged 40–74 years with one or more of long-term health conditions (e.g., diabetes (types 1 and 2), chronic obstructive pulmonary disease, asthma, coronary heart disease, heart failure, epilepsy, osteoporosis, with or without anxiety or depression)	Social isolation	- Face-to-face delivery - Contact with LWs can be face-to-face, via telephone, email and/or text messages - Individual sessions and group activities - Length of intervention: 4 to 14 months	*Ways to Wellness* provides a ‘hub’ model of social prescribing in which service users’ health and wellness goals are identified and where they are further connected to community and voluntary groups and resources such as welfare rights advice, walking groups, physical activity classes, arts groups, and continuing education. Promotion of volunteering opportunities and of improved self-care and sustained behaviour change related to healthier lifestyle choices are also encouraged
**(Nordin et al., 2020) **[57]**, Sweden**	- Community dwelling older adults aged 65 and over who receive home care to meet their social needs and/or experience loneliness (recruited from 2 municipalities)	Loneliness	- Face-to-face delivery - Group activities - Length of intervention: not stated	People with support from home care services were offered to go to community-based activity centers to receive support and increase their social participation
**(Todd, 2017) **[58]**, UK** **Unpublished PhD thesis**	- People aged 64–84 years self-identifying as lonely or socially isolated	Loneliness and social isolation	- Face-to-face delivery - Group sessions (*n* = 10) - Length of intervention: 10 weeks	*Museum-on-Prescription (MoP) programme* encourages participants to attend the museum once a week. MoP sessions consist of museum focused activities that may include museum object handling and discussion, guided visits to permanent displays and special exhibitions, creative writing and arts and crafts led by artists and museum staff
**(Van De Venter & Buller, 2015) **[59]**, UK**	- People with mild-to-moderate mental health problems (e.g., depression, social isolation, chronic conditions, etc.) - People experiencing commonly associated mental health challenges (e.g., social support and financial concerns)	Social isolation	- Face-to-face delivery - Group activities - Length of intervention: 20 weeks	*Arts-on-Referral (AoR) schemes* expose participants to a range of activities such as painting, textiles, music, photography and film. The schemes aim to improve participants’ wellbeing and social capital through collective engagement in creative arts, increased social contact and community engagement
**(Wildman et al., 2019) **[43]**, UK**	- People aged 40–74 years with one or more of long-term health conditions (e.g., diabetes (types 1 and 2), chronic obstructive pulmonary disease, asthma, coronary heart disease, heart failure, epilepsy, osteoporosis, with or without anxiety or depression)	Social isolation	- Face-to-face delivery - Contact with LWs can be face-to-face, via telephone, email and/or text messages - Individual sessions and group activities - Length of intervention: minimum 2 years	*Ways to Wellness* provides a ‘hub’ model of social prescribing in which service users’ health and wellness goals are identified and where they are further connected to community and voluntary groups and resources such as welfare rights advice, walking groups, physical activity classes, arts groups, and continuing education. Promotion of volunteering opportunities and of improved self-care and sustained behaviour change related to healthier lifestyle choices are also encouraged
**(Wood et al., 2021) **[60]**, UK**	- People aged 18 and over with multiple health conditions including co-existing physical and mental health concerns (e.g., depression, anxiety, social isolation - People with social needs associated with housing, benefits, and lack of support networks - People from an inner-city area of high socioeconomic deprivation	Social isolation	- Face-to-face delivery - Individual sessions and group activities - Length of intervention: not stated	Advice and services around health, employment and training were provided to participants. That includes health training (e.g., weight loss or health eating advice, alcohol or cigarette reduction and exercise advice), social cafés, benefits and housing advice, and volunteer work. There is no set pathway through the service and clients can access different services at different times in whatever order meets their needs
**(Woodall et al., 2018) **[61]**, UK**	- People aged 14 and over with physical and mental health difficulties (e.g., anxiety, social isolation, loneliness, etc.)	Loneliness and social isolation	- Face-to-face delivery - Group activities (up to 6) - Length of intervention: 16 weeks	*Wellbeing Coordinators* offer support to individuals and provide advice on local groups and services in their local community. The activities range from mental health and counselling advice, physical fitness classes, support for physical or emotional difficulties, finance and debt advice, and creative groups

### Quality of the studies

In terms of quality appraisal among peer-reviewed articles, none were scored under 5, nine articles scored between 6 and 7, and eight articles scored between 8 and 10 (see Table 2). Among the the non peer-reviewed records found in the grey literature, one scored 5 and the other scored 6. Overall, the Cohen’s kappa statistic value for quality appraisal was 0.79 indicating moderate inter-rater reliability [44].

### Thematic synthesis of results

Following a process of qualitative meta-synthesis of the 19 included articles, we identified three main themes.

Quotes are provided to illustrate each theme, accompanied by the socio-demographic characteristics of the individual (where available). Quotes given in italics are from participants in the original studies, and quotes not in italics represent the interpretations of the study authors. Table S1 identifies which studies contained data coded under each theme and subtheme, along with additional quotes.

### Theme 1: Increased sense of wellbeing

This theme relates to the enhanced feeling of wellbeing reported to result from the SP interventions investigated, and all articles alluded to this in some respect.

#### Subtheme 1: Decreased loneliness and social isolation

Fifteen articles (79%) conveyed that SP reduced feelings of loneliness and social isolation:

*“It made me feel less lonely. And coming out into places where there are quite a few other people … makes a place like a museum feel more familiar and that can’t be a bad thing.”* (Female, 65-69 years) [58]

After having been lonely for some time before the intervention, some participants described not feeling that way anymore because they were more aware of activities occurring in their local area and had the confidence to engage in them. Moreover, by sharing their similar experiences, they felt less lonely in facing challenges:

*“[…] hearing what other people are going through, makes you feel … better, or less isolated.”* (Female, 40-45 years) [59]

Others had learned to manage their time alone. One participant agreed with his home care worker that social contact with the service would be available if and when he needed it, reducing his feelings of loneliness when he was alone [57]. By being able to actively decide when to socially interact, he seemed to feel empowered to make decisions based on his actual needs.

#### Subtheme 2: Sense of belonging to the community

This theme centred on the strong sense of affiliation to a community experienced by participants and resulting from the SP interventions. Becoming a member of a community was meaningful as it provided greater support and a sense of belonging:

*“There is a sense of belonging in this room because we are all here together working. That sense of belonging carries out because people get up, talk, encourage each other. It’s a very nice feeling here.”* (Female, age not specified) [48]

Many commented that this feeling of belonging was only possible thanks to the groups and activities being perceived as a safe and non-judgemental spaces to engage in social interactions. Participants also emphasised the importance of feeling welcomed, as well as ensuring others felt comfortable in the shared space, fostering a sense of reciprocity:

*“I really felt a sense of belonging there [art hive] because I felt very welcomed.*” (Female, age not specified) [48]

“*Each time a person comes, we’re all happy to welcome the person. So, even if you don’t feel good and you come on Thursday, you feel great because it’s like people are waiting for you.*” (Female, age not specified) [48]

In some cases, this greater sense of belonging was generalised beyond the SP sessions:

“*[…] I like the town very much too. Well I mean I do feel part of it now and this course has helped me feel part of the society, very nice.”* (Male, 75-79 years) [58]

Being recognised as a valued member of a community provided participants with different types of support, from practical (e.g., transport, home help) to emotional and social support, which arose from both link workers and other people within the groups. However, some participants stated that a feeling of belonging was not contingent on forming close friendships in their groups. Having meaningful relationships was enough to promote their sense of connection to the community and increase engagement in the intervention:

“*I’ve gotten to know people I never knew before. So yes, they’re not my personal friends. We’re not doing things other than [the NORC Program activity], but we sometimes linger after the group and talk a little… Even if you don’t socialise elsewhere, the friendship feels good there.”* (Gender not specified, 60+ years) [52]

#### Subtheme 3: Improved self-confidence and self-worth

Participants clearly valued increased feelings of self-confidence and self-worth. Overall, being in a community with people facing similar experiences made participants feel more confident to talk about their issues. Link workers seemed to play an important role as they would generally help people to enter social situations and encourage them to raise questions “to gain the confidence needed to do it again by themselves in the future” [49]:

*“Well because I’ve been going further afield with [link worker], I feel more empowered to do better things and improve my life. I’ve got more confidence to do things.”* (Gender not specified, 44-84 years) [50]

Moreover, active engagement with the group provided motivation to attend the activities, which in turn engendered a sense of wellbeing:

*“After [my partner] passed away I was, not a recluse, but I just didn’t want to talk to anybody. But since I’ve been coming to see [the link worker] I’ve broadened my horizons and I get out … I’ve got a lot more confidence.”* (Male, 60-64 years) [42]

#### Subtheme 4: Sense of purpose, pride and achievement

Because some interventions involved the development of skills or represented a demanding task, some participants felt pride, achievement, and increased self-worth at having faced these challenges:

*“I’m doing something different. I’m achieving something in me [sic] old age that I didn’t think I’d be able to do*.” (Gender not specified, 50+ years) [51]

Again, link workers took an active part in fostering positive changes by considering participants’ preferences about which activities to attend and by encouraging them continuously to reach their objectives, giving rise to a sense of meaning and a sense of purpose as well as a feeling of achievement. Some spoke of looking forward to attending the activities and engaging with their community, which was often accompanied by the perception of being seen as “valuable members of society with a wealth of life experience to share” [53], increasing their feeling of self-worth:

*“I’d been feeling very depressed, I’ve been in the building trade for fifty years, very active, doing all my own repairs at home, I was a joiner. And then I’m suddenly stuck in a wheelchair. And it was more frustration […]. The service just gave me suggestions on things to do, like one thing I’ve always enjoyed is swimming. And I haven’t done it for years. And it was, you know, accessing things like that. There is a workshop where people go to do woodwork…I feel a bit better in myself knowing that there are things out there that I can do.”* (Male, 50+ years) [61]

#### Subtheme 5: Providing a distraction

In a few cases, being involved in the community and taking part to the activities was an opportunity for distraction from personal issues:

*“When I have social things to do, it helps with the other stuff. Sometimes when you’re just so focused on your issue, you don’t have time to recuperate, […], and this break just gives you an opportunity to just let go and unwind. It’s just good overall to escape for a minute and kind of give you clarity on what’s going on, […]. It’s a welcomed break.”* (Gender not specified, 18–81 years) [45]

### Theme 2: Factors that engendered an ongoing desire to connect with people

This theme relates to the realisation, following engagement in the SP intervention, that newly-formed relationships were valuable. The sense of reward experienced from making these connections engendered a desire to sustain them. For some participants, SP sessions represented their only social contact, and they expressed a strong desire to maintain social interactions. The eagerness to interact grew as participants built trusting relationships with their link workers and others from their activity groups:

*“It’s like you feel when you’re going to meet a friend. Coming here is enjoyable and I look forward to it.”* (Female, age not specified) [48]

One important component was the pleasure found in companionship, which created a strong sense of friendship that sometimes led to further interactions outside the SP setting. Several participants indicated that these relationships served as substitute family relationships, as they understood each other in a way family members could not.

*“I made some very, very good friends at NORC, and I’m 87 years old, and you figure when you’re in your eighties and you make new friends that become like sisters to you, I mean, it’s remarkable because you lose friends. You don’t expect to make friends.”* (Gender not specified, 60+ years) [52]

In one analysis [53], participants felt they were losing meaningful interactions with their family due to the constant presence of technological devices in the background, or avoiding them due to tensions over parenting matters with children and grandchildren. These factors led to preferences for socialising with their contemporaries encountered via SP:

*“They are better than family…”* (Gender not specified, 80 years) [53]

Nevertheless, significant relationships were also applicable to link workers, who were particularly appreciated for their listening skills and supportive and non-judgmental approach.

*“I look at him [link worker] as like a pal. It’s as simple as that.”* (Male, 65-69 years) [43]

*“She [link worker] was very friendly…She was there to just, generally, talk to. Like, a female companion type thing, because I've got none of that at home, it’s all males […] she’s just so friendly. We used to have a laugh, I would talk about my family, she would talk about hers. It wasn't as though she was like a worker, you know what I mean? It was that good.”* (Female, 55–59 years) [43]

Consequently, participants had become more likely to engage within their community and with people beyond the SP intervention:

*“It’s that being able to talk to somebody, and somebody willing to listen, I think that’s the crux of it, and not being judgmental.”* (Male, under 50 years) [61]

To forge these friendships within their activity groups, some participants identified the importance of sharing common interests and similar experiences. Personal stories were more easily shared in a relaxed and supportive atmosphere, and participants were more likely to engage in the activities:

*“It was interesting to meet other people in similar situations to me. To hear about their experiences and what they’d been through. How they were dealing with things.”* (Gender not specified, 18-70 years) [49]

Not having common interests with peers was perceived as isolating, so finding people with shared interests made socialising more enjoyable:

“*My interests are different from most people, so I tend to isolate myself a little bit. So, it was good for me in that sense because it got me out, it got me socialising, it got me even back into doing some artwork*.” (Female, age not specified) [48]

### Theme 3: Drawbacks perceived in SP

A few studies described perceived drawbacks of SP that had apparently prevented participants from gaining the benefits described by others.

Two studies [49, 52] noted that the desire to connect was lessened when people didn’t share similar interests or when they felt “their social needs were already being met through other channels, such as through their churches” [52]. Not being interested in the proposed activities or not sharing interests with others decreased the likelihood of continuing participation:

“*Maybe I just didn’t really feel like I fitted it in. I didn’t feel like the people I was around were really my age or people that I’d really have a social life with*.” (Gender not specified, 18-70 years) [49]

For another participant, not wanting to make “*any longer-term connections*” influenced the extent to which he had taken part in the programme [58]. In another study, feeling in a “*lower place*” emotionally was a barrier to engaging with the link worker and thereby getting involved within the community [49].

Additionally, where relationships with link workers or other participants were perceived as negative this created resistance to ongoing engagement with the programme:

“*I don’t like the people that go there.*” (Gender not specified, 60+ years) [52]

“*I just didn’t like the atmosphere at all … I think they [the staff] were impatient and I think with very elderly people, you’ve got to be really patient*.” (Female, 65-74 years) [55]

Another participant stopped participating in the programme because the link worker was perceived as over-enthusiastic in pushing them too rapidly [49]. Therefore, the initial contact with the link worker seemed important in establishing an alliance for the development of the relationship.

## Discussion

This meta-synthesis of 19 analyses of 18 qualitative datasets exploring the experiences of participating in SP to address loneliness and/or social isolation identified three main themes. Our first theme relates to the increased sense of wellbeing provided to the participants. Indeed, following their participation in SP programmes, many participants said that they experienced a reduction in loneliness and/or social isolation as well as improvements in several aspects of their wellbeing such as self-confidence and self-worth, a sense of belonging to a community, a way to distract themselves from their problems, and a sense of purpose, pride, and achievement. Our second theme specifically describes the factors that engendered a desire to reinforce connections with other people, both within and outside the SP programme, which in turn likely contributed to the reducing of loneliness and/or social isolation. Our third theme brought out the difficulties and potential harms some participants encountered in some SP programmes, whether due to inappropriate choice of SP activities, mismatches between an individual and the approach of their link worker, or being too unwell to take part. For instance, because participants didn’t feel their interests were catered for locally, they might have not engaged in the intervention and remained lonely and/or isolated with the associated mental and physical health implications.

The newly formed relationships with either the link worker or other members of activity groups, or both, were often perceived as rewarding, which motivated participants to continue interacting with other people, including those beyond the programme. Where participants had been in contact with link workers prior to the group activities, they generally spoke well of them, even if they had at first been cautious about participating.

### Findings in the context of other studies

To our knowledge, this review is the first meta-synthesis of qualitative studies describing the perceived benefits and drawbacks in the use of SP to address loneliness and social isolation. Our findings suggest that participants referred for SP recognise benefits in addressing their loneliness and/or social isolation. Viewed in the context of the wider literature, our data corroborate previous reviews that include both qualitative and quantitative findings [28, 62, 63] and a recent mixed methods primary study in which people taking part in SP schemes experience improved wellbeing, quality of life, social networks, and self-confidence, as well as reduced loneliness and social isolation [64]. However, it might be hard to conclude that SP is effective in reducing loneliness and social isolation since the SP interventions offered differ. Therefore, we believe SP might be a relevant tool for introducing loneliness and social isolation interventions, whose effectiveness will depend on the interventions themselves.

Our analysis implies that people felt more confident to interact in a group of people sharing similar interests and with common experiences. Previous evidence from a systematic review describing the effectiveness and acceptability of SP interventions reported increases in self-esteem and self-confidence as key outcomes of SP [62]. A population-based observational study found that social anxiety directly predicted loneliness, suggesting that high levels of social anxiety might lead to the avoidance of social contact that could otherwise reduce loneliness [65]. Pulling these findings together, a mechanistic pathway might be hypothesised that by increasing people’s confidence in social situations and allowing them to practise social skills in safe and welcoming environments, SP might reduce social anxiety, meaning people may be less avoidant of social situations and less isolated, which could lead to reduced loneliness. However, this would require testing in a rigorous mechanistic study.

Our findings also indicate that SP provides opportunities to expand social networks, which might help where loneliness arises from social isolation. However, for people whose loneliness does not stem from being objectively isolated, SP might have less of a beneficial effect. In such cases, a one-to-one intervention for a protracted period may be beneficial prior to joining a group setting. SP may provide the opportunity to combine this in a two-stepped approach, (i) individual interaction with a link worker and (ii) group interaction via referral to community activities. However, this approach can only work effectively if SP link workers and clients are allowed the flexibility to decide the number of sessions they need to maximise the benefits.

Furthermore, the findings from this review also point to the importance of the role of the link worker, with service users referring to link workers as a ‘pal’ or somebody ‘willing to listen’. This finding suggests that their role is not solely to encourage ‘behaviour change’ but to model the creation of a ‘relationship’ based on trust and empathy, where service user and link worker co-design a solution and interact on an equal footing. Other authors have adopted a range of psychologically-based conceptual frameworks to analyse the interaction between service users and link workers, including self-determination theory [66], social cognitive theory [67], transtheoretical model of behaviour change [67], and social identity theory [68]. From a conceptual perspective, it also appears important to consider psychological theories that focus away from instigating ‘behaviour change’ to building a ‘relationship’ between link worker and service user. This includes the concept of salutogenesis, with its emphasis on ‘generalised resistance resources’ [69] or the similar but more recent concept of ‘social scaffolding’ [70].

Some participants described drawbacks and potential harms in their experiences of SP services, including not sharing interest in the activities with other participants, dealing with burdensome health issues, and not liking the link workers. A recent mixed-methods study investigating the effect of SP on wellbeing and primary care utilisation had similar findings [71]. Participants did not benefit from SP interventions where they felt overwhelmed by other health needs, where the activities were not as expected, or if they met logistical problems attending the activities [71].

Overall, our synthesis of qualitative data from adults aged 18 to 95 years suggests that SP schemes are perceived as helping to reduce loneliness and/or social isolation. There is clearly a need for further qualitative studies to explore their acceptability in a range of age and ethnic groups. The varied nature of SP interventions means that RCTs on SP specifically may not be straightforward. Once robust evidence-based interventions for loneliness and/or social isolation have been developed through powered RCTs, SP might be one way of implementing these.

### Strengths and limitations

This meta-synthesis used a comprehensive search strategy to distinguish studies across a range of countries. We followed established guidelines and used a multidisciplinary team approach as well as lived experience involvement to plan the searches and conduct the analysis and synthesis. Although the first reviewer primarily conducted the literature searches, identification of pertinent studies, synthesis of themes and critical appraisal, a second reviewer independently coded 20% of studies for the screening process and the quality appraisal, and checked 20% for synthesis of themes, with a moderate to strong level of agreement. Every coding decision was subject to iterative discussion with the multidisciplinary team, paying attention to reflexivity, and this enhanced the validity of our findings. All findings were presented in the context of methodological quality.

The socio-demographic characteristics of study samples in this review represent limitations. The predominance of older participants in included samples limits the resonance of these findings to young and mid-life adults. This identifies a gap in the literature particularly given the high prevalence of loneliness in young people [72]. The high proportion of older adults also made it harder to explore age patterning of themes in experiences of SP. Additionally, most participants were female, which raises the question of whether women are more likely to seek help to address their loneliness, more attracted to the SP approach, more vulnerable to loneliness, or more willing to take part in research. This higher prevalence of women was also observed in another systematic review on the effectiveness of social prescribing programs in the primary care context [73]. Therefore, future research must aim to include more balanced samples in terms of gender, ethnicity and other demographic characteristics and to examine barriers to specific groups’ participation. Additionally, it was difficult for the reviewers to disentangle whether SP influenced social isolation or loneliness as many studies did not differentiate them clearly.

There was substantial heterogeneity in the interventions described, with wide variation in the length of programme (between 8 weeks and 3 years) and in the types of activities the SP interventions proposed, ranging from arts activities to community gardening and from health-related support groups to luncheon clubs. It therefore remains unclear what the active ingredients are in these approaches, and how long they should be delivered for. In addition, only three of the articles [43, 52, 58] included follow-ups, to convey perceptions of whether any benefits or drawbacks of participating had lasting effects. Finally, our search was for studies in English or French but only yielded English studies, which might lead to some important research findings having being missed.

### Implications for practice

Our findings are of relevance to clinicians and policymakers who may be considering SP for specific patient groups, identifying perceived benefits and drawbacks. These suggest that SP interventions must be carefully tailored to individuals’ needs and interests. This applies both to those referring into SP schemes and to link workers, who require time and flexibility to support individuals in their choices and consider the range and number of local community services available to each individual. Gaining early and ongoing feedback from participants would determine whether their expectations are being met, and whether they feel they connect with other participants. If not, changes could be implemented such as considering other local SP options. In some cases, link workers could help address anticipatory anxiety by conducting one-to-one sessions in preparation for group activities offered, and accompany individuals to the first activity. One paradigm for this was a SP service in Redbridge aimed at reducing social isolation, where link workers worked with clients on a one-to-one basis throughout the 12-week period to ensure their needs were being met, such as chaperoning, research and language support [74]. Greater awareness by referrers and link workers of participants’ preferences and concerns has the potential to improve acceptability.

From a public health perspective, specific ethnic groups, specific age groups, and socio-economically disadvantaged groups may suffer inequitable access to SP interventions or to local primary care services [75, 76] due to the inverse care law [77]. There is a higher prevalence of loneliness among people living in deprived areas [78]. It is therefore important that SP is provided equitably, perhaps at access points beyond GP surgeries, using community leaders as a means of addressing these inequalities.

### Future research

Although our findings support the acceptability and perceived positive effects of SP in reducing loneliness and social isolation among adults, the composition of samples means that these findings may only be generalisable to older females in Western settings. Further investigation into the experiences of specific socio-demographic groups when accessing and using SP is required. Future research should have a specific focus on loneliness across the life course and investigate how it is conceptualised in younger age groups [79]. The clear lack of ethnic diversity in our samples also needs to be addressed by conducting studies with different cultural groups to get a more comprehensive picture.

Obtaining link workers’ and family caregivers’ perspectives through qualitative research might also expand our knowledge of which strategies have the most benefits for whom. Additionally, more research could be conducted on the underlying cognitive and social mechanisms that cause SP to be perceived as beneficial for people experiencing loneliness and/or social isolation. We suggest isolating the impact of link workers from the impact of the community activities setting to understand their relative contribution. Alternatively, participant observation might be a way to understand how SP is delivered and determine what could be done better [80].

A potential next step might be well-designed controlled studies to assess whether SP programmes are effective tools for delivering interventions for reducing loneliness and social isolation, accompanied by qualitative research which enables a more in-depth examination of social prescribing themes. Where RCT design is problematic, for example in areas where SP constitutes ‘usual care’, alternative approaches such as cluster randomised controlled designs, or stepped wedge designs might be preferred, or the analysis of observation data using propensity scores. It would also be important to consider that RCTs may be examined in a realist evaluation framework [81]. Such a ‘realist RCT’ may be used to answer questions about what works, for whom, and in what circumstances. This would uncover not just intervention effectiveness but also the mechanisms and the contexts that drive practical implementation of SP for people experiencing loneliness and social isolation.

## Conclusion

Our meta-synthesis identified 19 relevant qualitative analyses of 18 datasets describing adults’ positive and negative experiences of SP to address their loneliness and/or social isolation. Findings suggest that some individuals experienced not only a perception of reduced loneliness and social isolation, but also a sense of increased wellbeing. Furthermore, meaningful relationships engendered their desire to connect. Negative aspects of the identified SP interventions included being uninterested in the proposed activities, having negative relationships with link workers, and having other priorities. This suggests a need for more person-centred SP, with greater choice of SP interventions. A flexible two-step approach combining an individual interaction with the link worker and then with other service users in a group setting, depending on the needs and aspirations of each service user, could therefore be the ideal model. To complement the findings of this meta-synthesis summarising acceptability, we need controlled studies describing the effectiveness of SP in tackling loneliness and social isolation, to meet the needs of policymakers.

## Supplementary Information

**Additional file 1: Supplementary Material 1.** Full search strategy.

**Additional file 2: Table S1.** References for themes and subthemes, and additional quotes.

## Data Availability

All data generated or analysed during this study are included in this published article [and its supplementary information files].
