# Supplementary material for: Do people perceive benefits in the use of social prescribing to address loneliness and/or social isolation? A qualitative meta-synthesis of the literature
Source: BMC Health Serv Res. 2022 Oct 19;22:1264. doi: 10.1186/s12913-022-08656-1 (PMC9580419; doi:10.1186/s12913-022-08656-1)
Supplement: Supplementary file 1 — Additional file 1: Supplementary Material 1. Full search strategy. [file 12913_2022_8656_MOESM1_ESM.docx]

## Supplementary material 1: Full search strategy

For all peer-reviewed databases:

Scopus:

ALL (( "Qualitative research"  OR  "qualitative stud*"  OR  interview  OR  "focus group"  OR  "semi-structured"  OR  unstructured  OR  "lived experience"  OR  ethnograph*  OR  "group discussion"  OR  "free text" OR narrative OR IPA OR "grounded theory" OR discourse* OR thematic*)  AND  ( "social prescribing"  OR  "social prescri*"  OR  "community refer*"  OR  "nature prescription"  OR  "connection prescription"  OR  "outdoor prescription"  OR  "well-being-co-ordinat*"  OR  "wellbeing-co-ordinat*"  OR  "well-being- coordinat*"  OR  "wellbeing-coordinat*"  OR  "community-navigat*"  OR  "community navigat*" )  AND  ( "social isolat*"  OR  lonel* OR “perceived social support” OR “perceived social isolat*” OR “emotional isolat*”))

Web of science:

ALL=(('social  isolat*'  or  lonel* or ‘perceived social support’ or ‘perceived social isolat*’ or ‘emotional isolat*’)  AND  ('social prescribing' or 'social prescri*' or 'community refer*' or 'nature prescription' or 'connection prescription' or 'outdoor prescription' or 'well-being-co-ordinat*' or 'wellbeing-co-ordinat*' or 'well-being-coordinat*' or 'wellbeing-coordinat*' or 'community-navigat*' or 'community navigat*')  AND  ('Qualitative research' or 'qualitative stud*' or interview or 'focus group' or 'semi-structured' or unstructured or 'lived experience' or ethnograph* or 'group discussion' or 'free text' or narrative or IPA or discourse* or thematic* or ‘grounded theory’)

Medline & PsycInfo & Embase (via Ovid): (*Social isolat* OR lonel* OR perceived social support OR perceived social isolat* OR emotional isolat*’) AND (Social prescribing OR social prescri* OR community refer* OR nature prescription OR connection prescription OR outdoor prescription OR well-being-co-ordinat* OR wellbeing-co- ordinat* OR well-being-coordinat* OR wellbeing-coordinat* OR community-navigat* OR community navigat**) *AND (Qualitative research OR qualitative stud* OR interview OR focus group OR semi-structured OR unstructured OR lived experience OR ethnograph* OR group discussion OR free text OR narrative OR IPA OR discourse* OR thematic* OR grounded theory)*

For non peer-reviewed databases:

- **Networked Digital Library of Theses and Dissertations (NDLTD)**: *("social prescribing" or "social prescription" or "community referral") AND ("loneliness" OR "lonely" OR "social isolation*")*
- **King’s Fund Library’s website**: *social prescribing AND social isolation*
- **Nuffield Trust’s website**: *("loneliness" or "social isolation") AND social prescribing*
- **Google Scholar**: *(social prescribing or community referral) AND (loneliness or social isolation)*
