# Supplementary material for: Do people perceive benefits in the use of social prescribing to address loneliness and/or social isolation? A qualitative meta-synthesis of the literature
Source: BMC Health Serv Res. 2022 Oct 19;22:1264. doi: 10.1186/s12913-022-08656-1 (PMC9580419; doi:10.1186/s12913-022-08656-1)
Supplement: Supplementary file 2 — Additional file 2: Table S1. References for themes and subthemes, and additional quotes. [file 12913_2022_8656_MOESM2_ESM.docx]

## Table S1: References for themes and subthemes, and additional quotes

| Themes and subthemes | Studies related to the themes | Additional quotes (where available) |
| --- | --- | --- |
| 1. Increased sense of wellbeing | (Alliance for Healthier Communities, 2020; Blickem et al., 2013; Cheetham et al., 2018; Fortune et al., 2021; Frerichs et al., 2020; Giebel et al., 2020; Greaves & Farbus, 2006; Greenfield & Mauldin, 2017; Hemingway & Jack, 2013; Kellezi et al., 2019; Kharicha et al., 2017; MacLeod et al., 2016; Moffatt et al., 2017; Nordin et al., 2020; Todd, 2017; Van De Venter & Buller, 2015; Wildman et al., 2019; Wood et al., 2021; Woodall et al., 2018) | *“Going there [art gallery] had positive effects for my mental health […].”* (Gender not specified, 18–81years) (Alliance for Healthier Communities, 2020) |
| *1.1. Decreased loneliness and social isolation* | (Alliance for Healthier Communities, 2020; Cheetham et al., 2018; Fortune et al., 2021; Frerichs et al., 2020; Giebel et al., 2020; Greaves & Farbus, 2006; Hemingway & Jack, 2013; Kellezi et al., 2019; Moffatt et al., 2017; Nordin et al., 2020; Todd, 2017; Van De Venter & Buller, 2015; Wildman et al., 2019; Wood et al., 2021; Woodall et al., 2018) |  |
| *1.2. Sense of belonging to the community* | (Alliance for Healthier Communities, 2020; Blickem et al., 2013; Cheetham et al., 2018; Fortune et al., 2021; Frerichs et al., 2020; Giebel et al., 2020; Greaves & Farbus, 2006; Greenfield & Mauldin, 2017; Hemingway & Jack, 2013; Kellezi et al., 2019; Kharicha et al., 2017; MacLeod et al., 2016; Nordin et al., 2020; Todd, 2017; Van De Venter & Buller, 2015; Wildman et al., 2019; Wood et al., 2021; Woodall et al., 2018) | *“There is a sense of belonging in this room because we are all here together working. That sense of belonging carries out because people get up, talk, encourage each other. It’s a very nice feeling here.* ”(Female, age not specified*)*(Fortune et al., 2021) |
| *1.3. Improved self-confidence and self-worth* | (Alliance for Healthier Communities, 2020; Cheetham et al., 2018; Frerichs et al., 2020; Giebel et al., 2020; Greaves & Farbus, 2006; Kellezi et al., 2019; Moffatt et al., 2017; Todd, 2017; Wildman et al., 2019; Wood et al., 2021; Woodall et al., 2018) | *“Anything like this sort of things or going to talk to people, it helps lonely people, helps with confidence as well and I think that’s the other thing with being lonely or on your own … you haven’t got the confidence to go in on your own.”* (Male, 75-79years) (Todd, 2017)  *“I guess in groups I feel uncomfortable around I talk less, but I felt fine around these people, they seemed to be very nice and approachable, and I ended up talking a lot more because of it.* (Gender not specified, 18-70years) (Frerichs et al., 2020)  *“It’s [social cafe] building my confidence up great. I’m making loads of friends. I mean, I’m in a craft group but I don’t really do much crafting when I’m, it’s more chatting and helping the others, so it’s lovely, and they’re just so friendly.”* (Gender and age not specified) (Wood et al., 2021) |
| *1.4. Sense of purpose, pride, and achievement* | (Alliance for Healthier Communities, 2020; Blickem et al., 2013; Fortune et al., 2021; Frerichs et al., 2020; Greaves & Farbus, 2006; Hemingway & Jack, 2013; MacLeod et al., 2016; Nordin et al., 2020; Todd, 2017; Van De Venter & Buller, 2015; Woodall et al., 2018) |  |
| *1.5. Providing a distraction* | (Blickem et al., 2013; Fortune et al., 2021; Van De Venter & Buller, 2015) | *“Something to get my, keep my mind stimulated, something to do and I like museum and it sounded very interesting.”* (Male, 75-79years) (Todd, 2017) |
| 2. Desire to connect | (Alliance for Healthier Communities, 2020; Blickem et al., 2013; Cheetham et al., 2018; Fortune et al., 2021; Frerichs et al., 2020; Giebel et al., 2020; Greaves & Farbus, 2006; Greenfield & Mauldin, 2017; Hemingway & Jack, 2013; Kellezi et al., 2019; Kharicha et al., 2017; MacLeod et al., 2016; Moffatt et al., 2017; Nordin et al., 2020; Todd, 2017; Van De Venter & Buller, 2015; Wildman et al., 2019; Wood et al., 2021; Woodall et al., 2018) | *“Friendship is important of course, meeting people is important. The art provides a means to share all of that.”* (Female, age not specified) (Fortune et al., 2021)  *“I started coming to this art hive and they told me there is another art hive . . . but I find it interesting coming here because it’s more for the socialization than the art*.” (Male, age not specified) (Fortune et al., 2021)  *“It’s social too because I am not artistic. I’m really not… But you like to come here and meet together*.” (Female, age not specified*)* (Fortune et al., 2021)  *“So you get to know people every week you're there [community group] … I have to come or they will keep saying, “Are you coming next week? Are you doing this? Are you involved?” So other people are asking me will I be there. The friends that I've taken or whatever.” (Male, 55–59*years) (Wildman et al., 2019) |
| 3. Drawbacks perceived in SP | (Frerichs et al., 2020; Greenfield & Mauldin, 2017; Kharicha et al., 2017; Todd, 2017) |  |
